# ST2 and IL-33 in Pregnancy and Pre-Eclampsia

**DOI:** 10.1371/journal.pone.0024463

**Published:** 2011-09-16

**Authors:** Ingrid Granne, Jennifer H. Southcombe, James V. Snider, Dionne S. Tannetta, Tim Child, Christopher W. G. Redman, Ian L. Sargent

**Affiliations:** 1 Oxford Fertility Unit, Nuffield Department of Obstetrics and Gynaecology, University of Oxford, Oxford, United Kingdom; 2 Nuffield Department of Obstetrics and Gynaecology, University of Oxford, Oxford, United Kingdom; 3 Critical Diagnostics, San Diego, California, United States of America; Centre de Recherche Public de la Santé (CRP-Santé), Luxembourg

## Abstract

Normal pregnancy is associated with a mild systemic inflammatory response and an immune bias towards type 2 cytokine production, whereas pre-eclampsia is characterized by a more intense inflammatory response, associated with endothelial dysfunction and a type 1 cytokine dominance. Interleukin (IL)-33 is a newly described member of the IL-1 family, which binds its receptor ST2L to induce type 2 cytokines. A soluble variant of ST2 (sST2) acts as a decoy receptor to regulate the activity of IL-33. In this study circulating IL-33 and sST2 were measured in each trimester of normal pregnancy and in women with pre-eclampsia. While IL-33 did not change throughout normal pregnancy, or between non-pregnant, normal pregnant or pre-eclamptic women, sST2 was significantly altered. sST2 was increased in the third trimester of normal pregnancy (p<0.001) and was further increased in pre-eclampsia (p<0.001). This increase was seen prior to the onset of disease (p<0.01). Pre-eclampsia is a disease caused by placental derived factors, and we show that IL-33 and ST2 can be detected in lysates from both normal and pre-eclampsia placentas. ST2, but not IL-33, was identified on the syncytiotrophoblast layer, whereas IL-33 was expressed on perivascular tissue. In an in vitro placental perfusion model, sST2 was secreted by the placenta into the ‘maternal’ eluate, and placental explants treated with pro-inflammatory cytokines or subjected to hypoxia/reperfusion injury release more sST2, suggesting the origin of at least some of the increased amounts of circulating sST2 in pre-eclamptic women is the placenta. These results suggest that sST2 may play a significant role in pregnancies complicated by pre-eclampsia and increased sST2 could contribute to the type 1 bias seen in this disorder.

## Introduction

Pre-eclampsia is a common but complex disease which affects 2.5–3% of pregnancies and threatens the lives of both the mother and baby. The maternal syndrome is driven by a dysfunctional uteroplacental circulation and the release of pro-inflammatory trophoblast derived factors [1], causing an excessive maternal inflammatory response with associated endothelial dysfunction. This results in new hypertension and proteinuria and other features that characterise the disease [2]. Normal pregnancy is also associated with a vascular inflammatory response secondary to the presence of the placenta. It is not only of lower intensity but characterized by a type 2 immune bias in contrast to the type 1 bias of pre-eclampsia.

One marker for type 2 immune bias is ST2L. ST2 is a member of the IL-1 receptor super-family with four isoforms of which the best characterised are ST2L, the membrane bound form, and an alternatively spliced soluble ST2 (sST2) [3]. ST2L is expressed on immune cells such as mast cells, macrophages and some lymphocytes (NK cells and type 2 T helper cells) [4], [5], [6]. High levels of sST2 mRNA are found in alveolar epithelium and cardiac myocytes with lower levels in peripheral blood mononuclear cells and endothelium [7]. In venous and arterial endothelial cells its expression is increased by pro-inflammatory stimuli such as IL-1β, phorbol ester or TNF-α [8] and by activated Th2 cells [9].

IL-33 is the ligand for ST2 [10]. It is constitutively expressed in the nuclei of endothelium of both large and small vessels [11] and is widespread in mucosal epithelial surfaces, localised in cytoplasm as well as the nucleus suggesting dual function [12]. As a soluble factor it signals via ST2L and the IL-1Receptor accessory protein (IL-1RAcP) and induces Type 2 cytokines such as IL-4, IL-5 and IL-13 from immune cells [6], [10].

IL-33 is released after cellular necrosis, and it belongs to a family of molecules called ‘alarmins,’ which includes HMGB1 and IL-1α, which also have dual nuclear and cytokine functions [13]. Full length IL-33 (33 kDa) can be cleaved by caspase-1 into a shorter 18 kDa form, which is reported to be functionally inactive [14], [15]. In addition, secreted IL-33 function can be prevented by sST2, which acts as a decoy receptor. Circulating IL-33 is elevated in asthma, anaphylaxis, rheumatoid arthritis, inflammatory bowel disease and sepsis [16], [17], [18], [19]. Circulating sST2 is increased in asthma, sepsis, autoimmune diseases such as systemic lupus erythematosus, dyspnea and acute myocardial infarction [17], [20], [21], [22], [23]. Increased circulating sST2 may have a negative impact on disease outcome, for example, it reduces IL-33 mediated neutrophil recruitment to the site of infection in sepsis [24], and is associated with poor survival after acute myocardial infarction [25]. In the heart, ischaemia reperfusion injury and infarction are moderated by IL-33 which protects cardiomyocytes from apoptosis; the benefit is partially abolished by sST2 [26]. As pre-eclampsia has a similar pathology [27], sST2 and IL-33 may have a role in this disease, either as a primary cause or a downstream player in the regulation of the systemic inflammatory response. Maternal blood levels of IL-33 and sST2 have not previously been investigated in pregnancy or pre-eclampsia. This paper addresses these issues.

## Materials and Methods

### Subject recruitment and ethics statement

These studies were approved by the Oxfordshire Research Ethics Committee and written consent was obtained from each participant. Three study groups were recruited. To determine changes in sST2 and IL-33 associated with pre-eclampsia, a single blood sample (plasma samples were collected using EDTA anticoagulation tubes) was taken from women with pre-eclampsia (n = 20) who were matched for age (+/−4 years), parity (0, 1–3, 4+), and gestational age (+/−13 days) to normal pregnant women, and for age and parity to non-pregnant controls (Table 1). To determine the changes with gestational age in circulating concentrations of sST2 and IL-33, healthy women were recruited to the Oxford Pregnancy Biobank in the first trimester of pregnancy (11–13 weeks gestation), and further samples acquired during the second (20–22 weeks) and third trimesters (30–34 weeks) (characteristics recorded in Table 2). 15 women became pre-eclamptic, these were matched to normal pregnant women. Additional samples were taken from 13/15 of the women who developed pre-eclampsia when diagnosed. 5 of these 15 women had developed pre-eclampsia prior to the third trimester sample being taken. The other 10 women had a third trimester sample taken on average 20 days (range 6–39 days) prior to diagnosis. Blood samples from 15 matched, healthy, non-pregnant women were also taken. Plasma samples were taken into sodium citrate anti-coagulation tubes. Finally, to further study the effect of gestational age on sST2 levels an additional 30 women with pre-eclampsia were matched to normal pregnant women, therefore totalling 50 matched cases of pre-eclampsia (Table 3).

**Table 1:** Clinical characteristics of women with pre-eclampsia, normal pregnant women and non-pregnant women (n = 20).

	Non-pregnant	Normal Pregnant	Pre-eclamptic	
Age (years)	30.85 (5.38)	30.7 (5.79)	29.45 (5.53)	n.s.
Nulliparity	14/20	14/20	14/20	n.s.
Gestation at sample (days)		238.12 (35.63)	241.45 (32.45)	n.s.
Booking systolic BP (mmHg)		109.45 (13.04)	122.50 (14.36)	p<0.05
Booking diastolic BP (mmHg)		65.90 (9.96)	77.15 (14.83)	p<0.05
Max systolic BP (mmHg)		124.05 (9.64)	176.30 (17.54)	p<0.0001
Max diastolic BP (mmHg)		75.21 (8.53)	113.65 (12.42)	p<0.0001
Birthweight (g)		3408.45 (402.18)	2176.25 (1022.97)	p<0.001

Data are shown as mean (+/− standard deviation).

**Table 2:** Clinical characteristics of women with pre-eclampsia and normal pregnant women (n = 15) in the longitudinal study.

	Non-pregnant	Normal Pregnant	Pre-eclamptic	
Age (years)	28.60 (4.93)	28.00 (5.50)	27.93 (5.75)	n.s.
Nulliparity	14/15	14/15	14/15	n.s.
Gestation at delivery (days)		282.00 (6.11)	250.50 (33.59)	p<0.001
Booking systolic BP (mmHg)		109.80 (13.68)	115.67 (13.64)	n.s.
Booking diastolic BP (mmHg)		65.53 (8.59)	70.07 (11.18)	n.s.
Max systolic BP (mmHg)		125.89 (7.57)	160.33 (13.22)	p<0.001
Max diastolic BP (mmHg)		78.93 (7.25)	107.13 (8.67)	p<0.001
Birthweight (g)		3439.93 (495.84)	2792.79 (1107.13)	n.s.

Data shown are mean (+/− standard deviation).

**Table 3:** Clinical characteristics of women with pre-eclampsia and normal pregnant women (n = 50).

	Normal Pregnant	Pre-eclamptic	
Age (years)	31.43 (5.58)	30.80 (5.61)	n.s.
Nulliparity	39/50	39/50	n.s.
Gestation at sample (days)	231.6 (34.87)	223.1 (34.22)	n.s.
Booking systolic BP (mmHg)	105.7 (11.09)	120.9 (14.28)	p<0.001
Booking diastolic BP (mmHg)	64.53 (9.82)	73.68 (12.59)	p<0.01
Max systolic BP (mmHg)	125.8 (11.62)	175.80 (18.13)	p<0.0001
Max diastolic BP (mmHg)	77.56 (7.12)	125.30 (33.46)	p<0.0001
Birthweight (g)	3392.73 (392.76)	1967.00 (1012.75)	p<0.0001

Data are shown as mean (+/− standard deviation).

Pre-eclampsia was defined as the new onset of a diastolic blood pressure (BP) ≥90 mmHg on at least two occasions within 24 hours and new onset proteinuria ≥300 mg in a 24 hour urine collection, 50 mg/mmol protein/creatinine ratio or at least 2+ on dipstick testing on two consecutive measurements. All cases and controls had singleton pregnancies with no known fetal abnormality. Blood samples were collected, plasma and serum were isolated and the samples stored at −80°C until analysis.

There were no significant differences in age, parity or gestation in any of the groups. At the first trimester sample both systolic and diastolic blood pressure was significantly increased in women whose pregnancy was complicated by pre-eclampsia. It is well recognized that increased blood pressure at booking is a risk factor for the disease [28]. Case characteristics are detailed in tables 1, 2 and 3.

### Detection of sST2 and IL-33 by ELISA

sST2 was measured in plasma using the Presage® ST2 Assay (Critical Diagnostics, San Diego, USA) according to the manufacturer's instructions. IL-33 was measured in serum or plasma using a human IL-33 ELISA kit with pre-coated plates (BioLegend, San Diego, U.S.A.). We found that the same amount of IL-33 is detected in plasma or serum samples from the same individual (data not shown). For each assay both standards and samples were tested in duplicate.

### Detection of ST2 and IL-33 by immunohistochemistry

Tissue samples from normal (n = 3) and pre-eclampsia placentas (n = 3) obtained after caesarean section were fixed in 10% formalin and processed into paraffin blocks. Serial sections (5 µm) were cut on coated slides and dried. Slides were deparaffinised in Histo-Clear (National Diagnostics, MERCK, Eorulab S.A.), rehydrated in a graded alcohol series, and sodium citrate antigen retrieval performed. Sections were pre-blocked with 10% FCS/PBS for one hour at room temperature. Endogenous peroxidase was quenched with 3% hydrogen peroxide. The sections were incubated overnight at 4°C with 1 µg/ml of anti-ST2 antibody (clone 9F8, Critical Diagnostics, San Diego, USA), 1 µg/ml of anti-IL-33 antibody clone Nessy-1 (Enzo Life Sciences, New York, USA) or the same concentration of a negative control mouse IgG antibody (Dako, Denmark). A cytokeratin 7 antibody (Dako, Denmark) was used as a positive control to label the syncytiotrophoblast (data not shown). A biotin-free, tyramide signal amplification system was used (Dako, Denmark) for detection, following the manufacturer's instructions. The sections were finally counterstained with haematoxylin. Slides were examined using a Zeiss Axioskop brightfield microscope with a Micropublisher 5MP RT camera.

### Detection of ST2 and IL-33 by immunoblotting

Placental tissue samples (normal (n = 9) and pre-eclampsia (n = 9)) were lysed using HEPES lysis solution supplemented with Complete Mini protease inhibitor cocktail (Roche, Germany). Placental lysate protein concentrations were determined using a BCA protein assay kit (Pierce, Thermo Scientific, Illinios, USA). Proteins (30 µg) were resolved by 10% Bis-Tris Novex SDS-PAGE (Invitrogen, Paisley, UK) and transferred to PVDF membrane. Membranes were incubated for 45 minutes in blocking buffer (PBS with 5% BLOTTO (Santa Cruz, California, USA) and 0.1% Tween) at room temperature. The membranes were then incubated overnight with the appropriate primary antibody at 4°C. Primary antibodies used were goat anti-ST2 (0.5 µg/ml) (R&D Systems, Minneapolis, USA ), murine anti-IL-33 (0.1 µg/ml) clone Nessy-1 (Enzo Life Sciences, New York, USA) or murine anti-β-actin (0.155 µg/ml) (Abcam, Massachusetts, USA). Reactions were visualised by incubating the membranes with the appropriate anti-mouse or anti-goat secondary antibody conjugated to horseradish peroxidase (Dako, Denmark) for one hour and detected using an enhanced chemiluminescence system (Pierce, Thermo Scientific, Illinios, USA).

### Placental perfusion model

To determine if IL-33 or sST2 are released from the villous surface into the maternal circulation a modified dual placental perfusion system was used as previously described [29]. Placentas were obtained from normal pregnant, healthy women (n = 8) or women with pre-eclampsia (n = 5) undergoing elective caesarean section, without labor, and were processed immediately. The maternal perfusates were centrifuged to remove cellular debris in a Beckman J6-M centrifuge at 600 g for 10 min at 4°C, and aliquots of the supernatants frozen at −80°C until analysed. sST2 was measured in the perfusate by ELISA as described above, IL-33 was measured using a human IL-33 Duoset ELISA (R&D Systems, Minneapolis, USA).

### Release of sST2 from placenta explants

All placentas were from pregnant, healthy women undergoing elective caesarean section, without labor, and were processed immediately. Freshly delivered placentae (n = 3) were first rinsed in ice cold Hanks balanced salt solution and placed into a glove box maintained at 8% O_2_. Placental pieces, cut from undamaged lobules that appeared healthy, were rinsed in 8% O_2_ equilibrated ice cold explant culture medium (DMEM containing 10% foetal bovine serum (PAA Laboratories GmbH, Austria), 1% antibiotic and antimycotic solution (Sigma Aldrich, UK) and L-glutamine) before being placed into ice cold fresh equilibrated explant culture medium. Placental pieces of approximately 2 mm in diameter were then dissected and distributed equally between Costar Netwell (24 mm diameter, 500 µm mesh) supports in 6-well plates containing 4 ml/well equilibrated explant culture medium (10 explants/well). Placental explants were finally washed again with a medium change before being incubated under conditions ‘normoxic’ for trophoblast (8% O_2_/87% N_2_/5% CO_2_) [30] for 24 hours. Explants were also treated with 100 ng/ml TNF-α or 100 ng/ml IL-1β (Peprotech, NJ, U.S.A.) under normoxic conditions, placed at 1% O_2_/94% N_2_/5% CO_2_ to stimulate hypoxic conditions, or placed at 1% O_2_/94% N_2_/5% CO_2_ for 1 hour before switching to 8% O_2_/87% N_2_/5% CO_2_ for 23 hours, simulating hypoxia-reoxygenation conditions. After 24 hr the explant supernatants were collected and centrifuged at 600 g for 10 min to remove cell debris. Explants were rinsed in PBS, collected and lysed in HEPES lysis buffer supplemented with Complete Mini protease inhibitor cocktail (Roche, Germany) and protein concentration determined using a BCA Assay (Pierce, Illinois, U.S.A.). The quantity of sST2 released from placental explants was standardized to the protein concentration of the total explants per condition. Supernatants were collected and stored at −80°C for ELISA. Results were expressed as % sST2 release from explants under varying conditions compared to normoxia (100%). IL-33 was measured in the supernatants by ELISA (R&D Systems).

### Statistical analysis

Gaussian distribution of data was confirmed using a Kolmogorovv-Smirnov test. For the longitudinal study, where the data were normally distributed, a repeated measures ANOVA was used with a Bonferroni post hoc test. For non-parametric longitudinal data a Kruskal Wallace test was used with a Dunn's post hoc test. To assess the predictive value of sST2 for pre-eclampsia a ROC curve was generated.Where paired data were shown to be normally distributed, a paired t-test was performed. Non-parametric paired data was analysed using a Wilcoxon matched pairs test. Non matched data comparing non-pregnant to longitudinal data was compared with an unpaired t test. Statistics were generated using Prism and SPSS software. Values of p<0.05 were considered to be statistically significant.

## Results

### There is no significant variation in circulating IL-33 in women with normal or pre-eclamptic pregnancies

**Figure 1:**
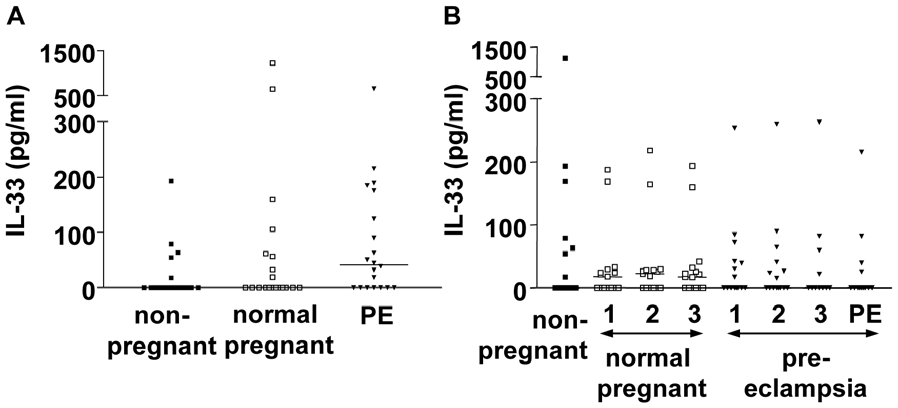
Circulating IL-33 in pregnancy and pre-eclampsia. A) IL-33 was detected by ELISA in the serum from women with a diagnosis of pre-eclampsia, matched controls who had a normal pregnancy and matched non-pregnant women (n = 20). No significant differences in IL-33 were identified. Bars represent median values. B) IL-33 was detected by ELISA in samples taken throughout pregnancy in women who developed pre-eclampsia and matched controls who had an uncomplicated pregnancy (n = 15). Blood samples were taken in each trimester of pregnancy (1, 2, 3) and at the diagnosis of pre-eclampsia (PE), and compared to non-pregnant matched women. There were no significant differences throughout pregnancy or between normal and pre-eclampsia pregnancies. Bars represent median values.

IL-33 serum levels were measured in 20 matched trios of women with pre-eclampsia, normal pregnant women and non-pregnant controls (Figure 1A). Despite a raised median value in the pre-eclamptic women there were no significant differences between groups. IL-33 was then measured in serum samples taken in the first, second and third trimester from 15 women who subsequently developed pre-eclampsia and 15 matched normal pregnant controls. An additional sample taken at the time of pre-eclampsia diagnosis was available for 13 of the women. There were no differences in IL-33 levels throughout pregnancy in women who went on to develop pre-eclampsia compared to the normal pregnant controls, or during the active disease (Figure 1B).

### sST2 is increased in the third trimester of normal pregnancy and is significantly elevated in pre-eclampsia prior to the onset of clinical symptoms

**Figure 2:**
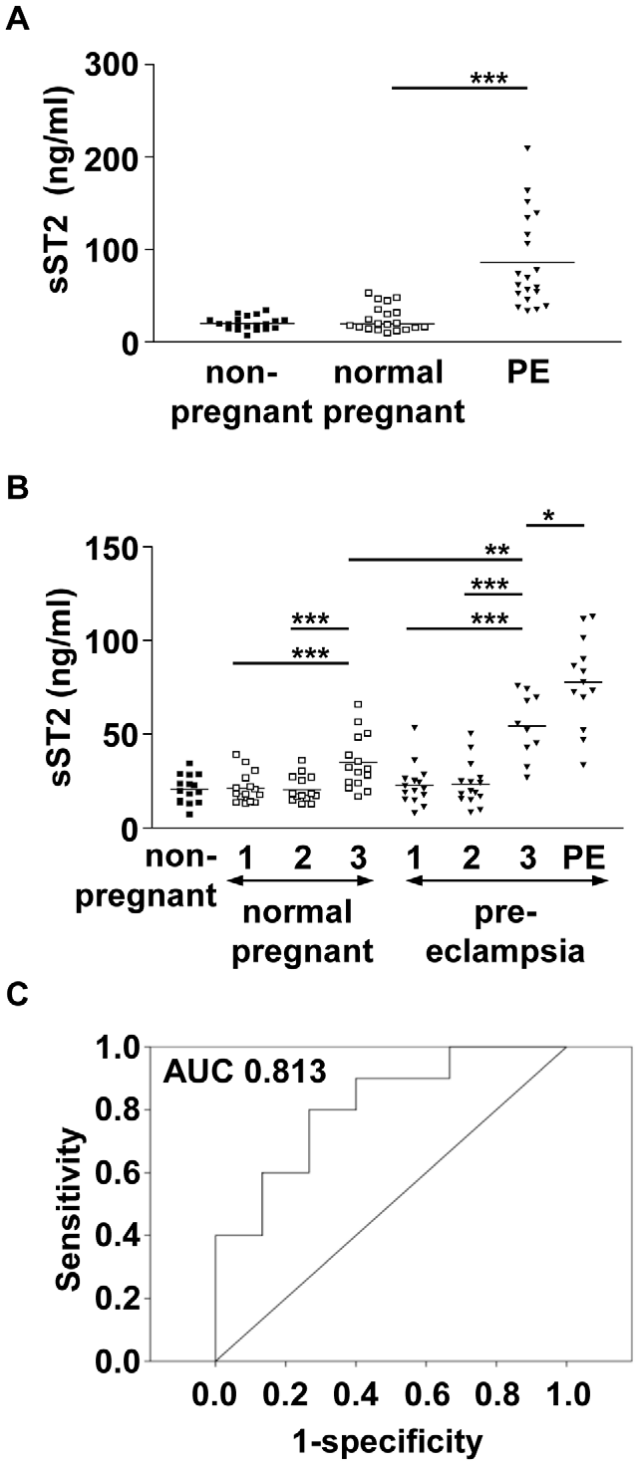
Circulating sST2 in pregnancy and pre-eclampsia. A) sST2 was detected by ELISA in the plasma from women with a diagnosis of pre-eclampsia, matched controls who had a normal pregnancy and matched non-pregnant women (n = 20). sST2 was significantly raised in pre-eclampsia compared to normal pregnancy (p<0.001). B) sST2 was detected by ELISA in samples taken throughout pregnancy in women who developed pre-eclampsia and matched controls who had an uncomplicated pregnancy (n = 15). Blood samples were taken in each trimester of pregnancy (1, 2, 3) and at the diagnosis of pre-eclampsia (PE), and compared to non-pregnant matched women. There is a significant increase in plasma sST2 in the third trimester of normal pregnancy compared to the first or second trimester (p<0.001), and compared to the non-pregnant group (p<0.001). sST2 was significantly raised in pre-eclampsia compared to normal pregnancy during the third trimester (p<0.01), prior to the onset of clinical symptoms. Levels further increased when pre-eclampsia symptoms presented (p<0.05). Bars represent mean values, * = p<0.05,** = p<0.01, *** = p<0.001. C) ROC curve analysis indicates sST2 levels in the third trimester sample (n = 10) are likely to predict pre-eclampsia (AUC = 0.813).

Circulating sST2 was next measured in 20 matched trios of women with pre-eclampsia, normal pregnant women and non-pregnant controls. sST2 levels were significantly increased (p<0.001) in women with pre-eclampsia (mean 85.89 ng/ml) compared to normal pregnant (mean 25.20 ng/ml) and non-pregnant women (mean 20.37 ng/ml) (Figure 2A). Plasma sST2 was measured in samples from normal pregnant and pre-eclamptic women throughout each trimester of pregnancy, with an additional sample taken at the time of diagnosis. There was a significant increase in sST2 in the third trimester of normal pregnancy (mean 34.87 ng/ml) compared to the first (mean 21.48 ng/ml) and second (mean 20.60 ng/ml) trimesters (p<0.001) (Figure 2B). Similarly, sST2 levels increased as pregnancy progressed, and levels were significantly higher in the third trimester of pre-eclamptic pregnancies (mean 54.28 ng/ml) compared to third trimester samples from normal pregnancies (p<0.001), even before the onset of clinical symptoms. When clinical symptoms were present sST2 levels were further increased (mean 77.82 ng/ml). The predictive value of sST2 at the third trimester sample time was assessed by generating a ROC curve, giving an AUC of 0.813, the 95% confidence interval had a lower bound of 0.642 and upper bound of 0.984. On average the women (n = 10) developed pre-eclampsia 20 days after this sample was taken. No differences between non-pregnant samples and normal pregnant first and second trimester samples were noted, however, as expected, there was a significant increase in the third trimester (p<0.001).

**Figure 3:**
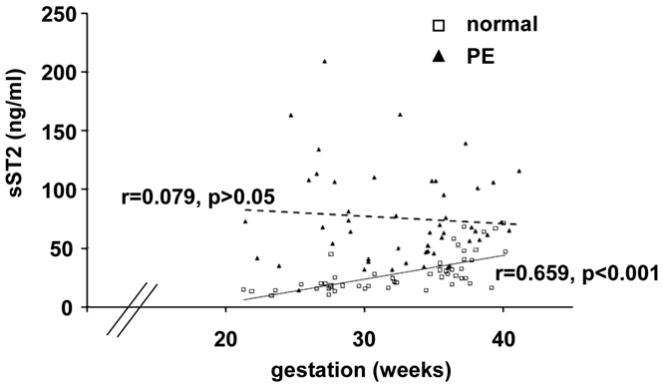
Comparison of sST2 levels to gestational age. A) sST2 was detected by ELISA in plasma from women with a diagnosis of pre-eclampsia and matched controls who had a normal pregnancy (n = 50). In normal pregnancy sST2 levels increase towards term. In pre-eclampsia sST2 levels are significantly increased compared to controls (p<0.001). Differences in sST2 levels in normal and pre-eclampsia pregnancies were more apparent in early onset disease.

This study was then extended to a larger group of 50 women with pre-eclampsia and matched normal pregnant women (Figure 3). The statistically significant increase in circulating levels of sST2 in the pre-eclampsia group was confirmed (p<0.001) (data not shown). Within the pre-eclamptic patient group the diagnosis was made between 22 weeks and 41 weeks gestation. sST2 levels were therefore compared to gestational age in both groups. In normal pregnancy circulating sST2 levels were strongly correlated with gestational age (r = 0.659, p<0.001) (Figure 3). In pre-eclampsia there was a poor correlation with gestational age (r = 0.079, p>0.05) as even in early onset pre-eclampsia sST2 levels were high.

### sST2 and IL-33 are expressed by both normal and pre-eclampsia placentas

**Figure 4:**
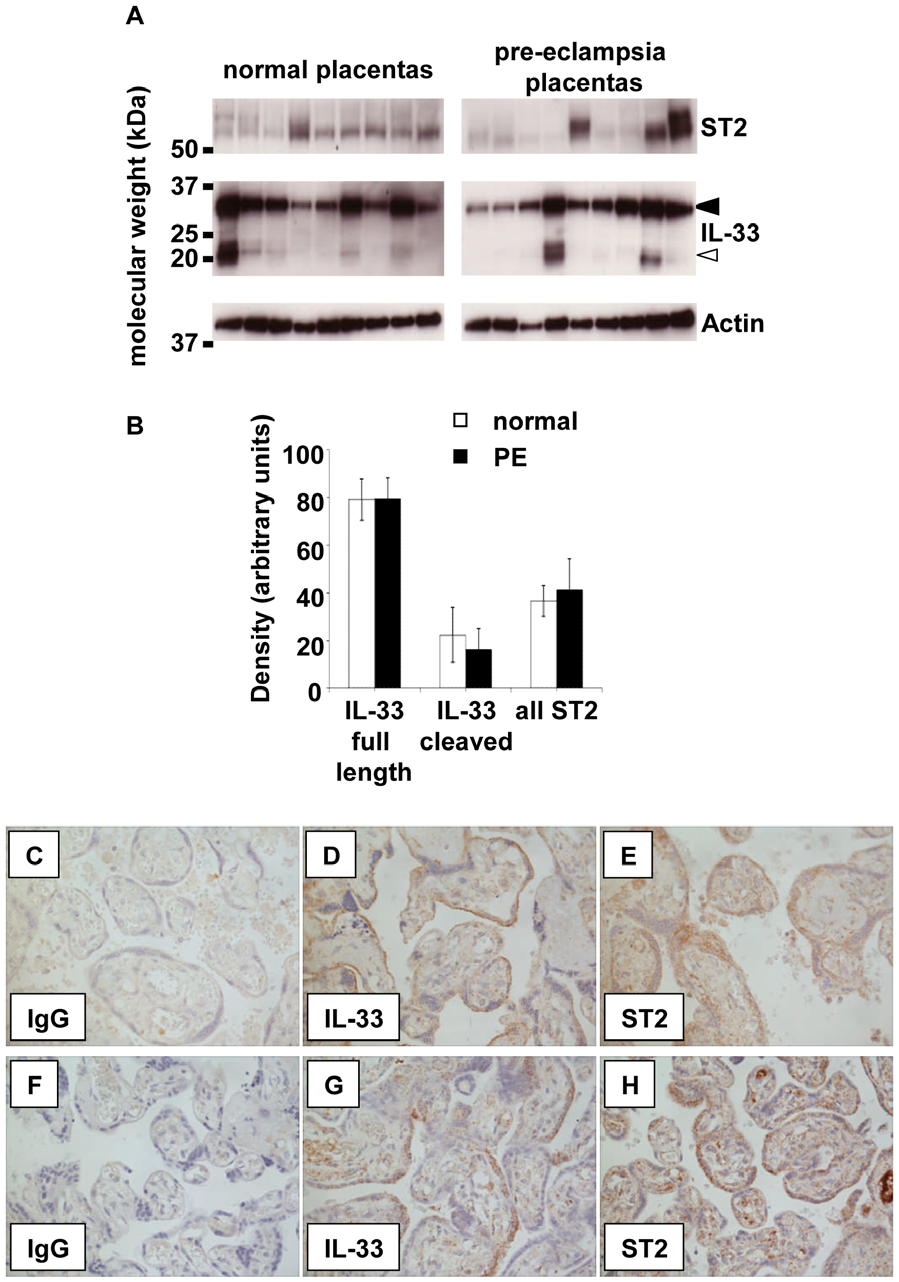
Expression of IL-33 and sST2 by the placenta. A) IL-33 was detected by western blotting of placental lysates from normal and pre-eclampsia placentas (n = 9). Both the full length (closed arrow) and cleaved forms (open arrow) were detected. ST2 was detected in each of the placental lysates. B) Densitometry of protein bands standardised to actin expression revealed no change in the levels of IL-33 nor sST2 between normal and pre-eclamptic placentas. C–H) Expression of IL-33 and sST2 can be seen in by immunocytochemical analysis of both normal (n = 3) and pre-eclampsia (n = 3) placentas (C-E and F-H show staining from two representative placentas respectively).

As circulating sST2 is increased in pregnancy, we speculated that the placenta may be a source. Although significant changes to circulating IL-33 were not detected throughout pregnancy it may be a local mediator that is not detected systemically, therefore we also investigated IL-33 expression. Immunoblotting showed the presence of IL-33 and ST2 in lysates of all normal and pre-eclampsia placental tissues studied (Figure 4A). Two forms of IL-33 were detected; full length IL-33 (closed arrow) was identified in all normal and pre-eclampsia placentas and the shorter cleaved form was identified in several of the placentas (open arrow). All of the normal and pre-eclamptic placentas expressed ST2. Densitometry was performed to standardise protein expression to actin quantity, no differences between normal and pre-eclamptic placentas were detected (Figure 4B). Placental sections were then analysed immunohistochemically to determine the cellular localisation of the IL-33 and ST2 (Figure 4C–H). Labelling for both molecules was variable. IL-33 localised to the syncytiotrophoblast and the chorionic villi. ST2 localised to some cells in the villous stroma with staining of the syncytiotrophoblast. No differences in staining profiles were seen between normal and pre-eclampsia placentas. No specific staining was seen in IgG controls and the positive control antibody (anti-Cytokeratin-7) strongly stained the syncytiotrophoblast (data not shown).

### The placenta may be the source of some of the sST2 in pregnancy and pre-eclampsia

**Figure 5:**
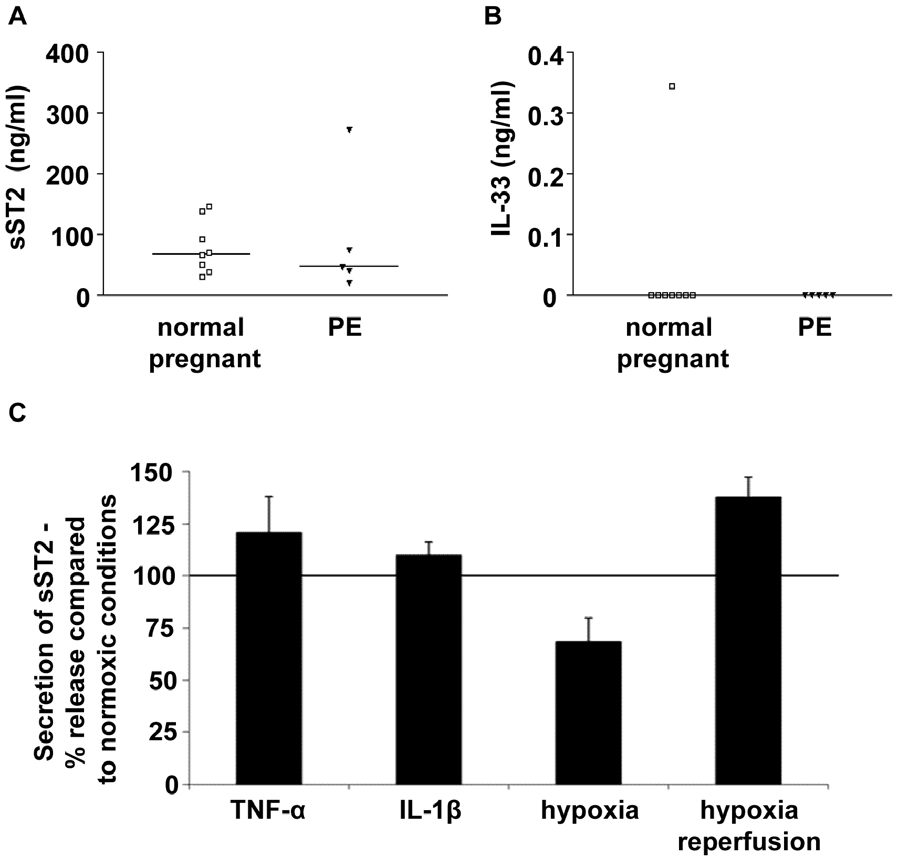
The placenta releases sST2. sST2 (A) and IL-33 (B) were detected by ELISA in maternal side perfusate from an ex vivo of placental perfusion model. Normal (n = 8) and pre-eclampsia (PE) (n = 5) placentas were perfused. sST2 could be identified in media from all of the normal and pre-eclampsia placental perfusions, IL-33 could be identified in one sample from a normal pregnancy. Bars represent median values. C) Placental explants release sST2, secretion was increased by treatment with TNFα and IL-1β (100 ng/ml) and hypoxia-reperfusion injury, and was decreased under hypoxic conditions (n = 3).

To determine whether sST2 and IL-33 can be released from the placenta into the maternal circulation, supernatants from the maternal side of placental perfusions (whereby the maternal blood flow across the syncytiotrophoblast is mimicked and maintained ex vivo) from normal (n = 8) and pre-eclampsia (n = 5) placentas were assayed. High levels of sST2 could be detected in all of the perfusates analysed from both normal and pre-eclampsia placentas (Figure 5A). IL-33 was detected by ELISA in the perfusate (Figure 5B) from only one normal placenta. To ensure that the ELISA is able to detect IL-33 in this system, and in the presence of sST2, we pre-incubated recombinant IL-33 (from the ELISA standards) with culture medium alone or maternal perfusion samples known to contain sST2 for 16 hours prior to ELISA (data not shown). IL-33 could still be detected under each of these conditions, suggesting that neither the media nor sST2 prevent detection by ELISA.

### sST2 secretion from the placenta is increased under conditions mimicking pre-eclampsia

Next we investigated placental release of sST2 and IL-33 changes when treated with pro-inflammatory cytokines, and hypoxia or hypoxia/reoxygenation conditions. Placental explants were treated for 24 hours with TNFα or IL-1β (100 ng/ml), sST2 secretion increased by 20.6 and 9.7% respectively (Figure 5C). Explants cultured under hypoxic conditions (1% O_2_) released 31.6% less sST2 than explants under normoxic conditions (8% O_2_). The greatest increase (37.9%) in sST2 levels was induced by hypoxia/reperfusion conditions (1 hour at 1% O_2_ followed by 23 hours at 8% O_2_). IL-33 could not be detected in the any of the explant supernatants.

## Discussion

sST2 levels were significantly increased in the third trimester of normal pregnancy and further increased in pre-eclampsia. These increases were detectable prior to the onset of clinical symptoms. Circulating sST2 is increased in a variety of conditions associated with systemic inflammation (summarised in [7]). Pre-eclampsia, as a systemic inflammatory disease secondary to an oxidatively stressed placenta, can now be included in this list.

The third trimester placenta expresses ST2, this confirms the previous report of Kumar et al (1997) [31]. Western blotting identified ST2 in all of the placental lysates analysed, no differences between groups were detected. Immunohistochemical staining identified ST2 in the syncytiotrophoblast layer, suggesting it could be secreted into the maternal circulation. Placental secretion of sST2 was first confirmed using a placental perfusion model. Perfusates from all of the normal and pre-eclampsia placentas examined contained sST2, although it was not possible to directly compare the concentrations of sST2 in each of the perfusates - the placentas were from varying gestational ages and the size of placental lobes perfused (and hence the area of syncytiotrophoblast contacted) was variable. sST2 is also produced by alveolar epithelial cells, cardiomyocytes and, to a lesser extent, by brain and small bowel cells [7], therefore the placenta is unlikely to be the sole source of sST2 in pregnant and pre-eclamptic women. In cardiac conditions associated with ventricular hypertrophy and remodelling (also a feature of pre-eclampsia [32]) increased circulating levels of sST2 are derived from cardiomyocytes activated by mechanical stress or pro-inflammatory stimuli [20]. Strong clinical evidence implicates the endothelium as an additional source [8], and ST2 is produced by proliferating endothelium in vitro [33]. sST2 is also increased in other conditions involving the endothelium such as Dengue Fever [34] or sepsis [17]. Endothelial dysfunction, a well known feature of pre-eclampsia [35] could also contribute to raised levels of sST2 in pre-eclampsia.

Placental explants from normal pregnancies also secreted sST2, and levels were increased in response to pro-inflammatory stimuli (IL-1β or TNF-α) or hypoxia-reoxygenation injury, both of which are features of pre-eclamptic placentas [1]. In other cell types sST2 secretion is stimulated by stress, particularly inflammatory stresses [31], and has anti-inflammatory actions [36] through its blockade of the danger signal delivered by IL-33. We have previously shown that expression of ST2L is increased on peripheral blood NK and NKT-like cells in pregnancy [37], which suggested that these might be a target for its ligand IL-33 and contribute to the type 2 bias of normal pregnancy. The production of sST2 may reflect the enhanced ‘type 1’ inflammatory environment of the disorder compared to normal pregnancy, as sST2 acts as a decoy receptor for IL-33, competing with membrane bound ST2L. Therefore, the very high levels of circulating sST2 seen in pre-eclampsia would be predicted to decrease IL-33 binding to its receptor, thereby contributing to the type 1 bias seen in this disease.

Here we can find no differences in circulating IL-33 between normal pregnant and non-pregnant women at any stage of gestation. Endothelial cells and epithelial cells constitute major sources *in vivo* [11], [36] and here we show the placenta also expresses IL-33. IL-33 does not have a signal peptide, so must be secreted non-classical pathways, such as after cell necrosis as an alarmin, or as a proinflammatory cytokine with a general role as an amplifier of innate immune responses [13], [38]. However, IL-33 also acts as an anti-inflammatory intracellular nuclear factor through binding to, and inhibition of, NF-κB [39], [40]. To what extent these functions are relevant to the normal pregnancy and the pathogenesis of pre-eclampsia remains to be elucidated.

Pre-eclampsia is thought to be caused by excessive release of anti-angiogenic sFlt-1 (the soluble VEGF receptor) from the oxidatively stressed placenta [41]. Angiogenesis is stimulated by, and stimulates, inflammation [42]. In general VEGF and sFlt-1 are upregulated together with vascular inflammation. sFlt-1, acting as a decoy receptor for VEGF, is anti-inflammatory, for example, higher circulating levels are associated with enhanced survival in experimental sepsis [43]. sST2 seems to be a second anti-inflammatory factor produced by the placenta in excess amounts during the course of pre-eclampsia. The links between the mechanisms by which the release of these two factors is increased in preeclampsia have yet to be clarified.

In summary this study shows for the first time that sST2 increases in the third trimester of normal pregnancy and that sST2 is significantly increased in pre-eclampsia probably secondary to the systemic vascular inflammation of the disorder. In addition we have shown that sST2 is raised in pregnancies destined to be complicated by pre-eclampsia even prior to the onset of clinical symptoms, which raises the possibility of its use as a novel biomarker for the disease, perhaps independent from other markers such as sFlt-1, endoglin and placental growth factor [44]. We have shown that the origin of at least some sST2 in pregnancy is the placenta and that a stressed placenta, as in pre-eclampsia, secretes more sST2. The functional role of sST2 in pregnancy and pre-eclampsia has yet to be established and is under investigation.
